# Condylar fracture location is correlated to exercise history in Thoroughbred racehorses

**DOI:** 10.1111/evj.14091

**Published:** 2024-04-07

**Authors:** Thomas C. Bergstrom, Mathieu Spriet, Ryan S. Carpenter, Kevin L. Jacques, Susan M. Stover

**Affiliations:** ^1^ William R. Prichard Veterinary Medical Teaching Hospital, University of California Davis California USA; ^2^ Department of Surgical and Radiological Sciences School of Veterinary Medicine, University of California Davis California USA; ^3^ Equine Medical Center Cypress California USA; ^4^ Present address: Equine Medical Center, Cypress, California USA

**Keywords:** disease, horse, joint, microfracture, osteochondral, palmar, remodelling

## Abstract

**Background:**

Condylar fractures are a major cause of morbidity and mortality in Thoroughbred racehorses. Condylar fractures have a variety of fracture configurations that suggest there may be differences in aetiopathogenesis.

**Objective:**

To determine if exercise history differs with condylar fracture location in a population of Thoroughbred racehorses.

**Study design:**

Retrospective analysis of clinical and exercise data.

**Methods:**

Exercise history of Thoroughbred racehorses that had condylar fracture repair between 1 January 2018 and 28 February 2021 was compared between racehorses that had fractures located radiographically either within the parasagittal groove (PSG) or abaxial to the PSG (non‐PSG). Age, sex, and last event (race, timed work) matched control groups were compared between the PSG and non‐PSG groups. Additionally, exercise history variables of both groups were each compared with a group‐specific control population, each consisting of three control racehorses of equivalent age and sex matched to each affected racehorse by last event (race or official timed work) before fracture.

**Results:**

Eighty‐two horses with 84 fractures (45 PSG, 39 non‐PSG) met inclusion criteria. Age was not different between groups (PSG: 3.4 ± 1.3 years [mean ± SD], non‐PSG: 3.7 ± 1.3, *p* = 0.3). Number of races (PSG: 5.3 ± 7.1, non‐PSG: 11.4 ± 8.9, *p* < 0.001), total race furlongs (PSG: 38.2 ± 54.7, non‐PSG: 79.2 ± 64, *p* = 0.003), and number of active days (PSG: 304 ± 224, non‐PSG: 488 ± 314, *p* = 0.003) before fracture were greater; while mean number of layups was fewer (PSG: 1.0 ± 1.2, non‐PSG: 0.5 ± 0.7, *p* = 0.02) in horses with non‐PSG fracture. Horses with non‐PSG fracture had more differences compared with their respective control group than horses with PSG fractures. Outcomes following fracture repair were not different between groups.

**Main limitations:**

Retrospective study, one regional racehorse population, two‐dimensional imaging and potential inherent bias for fracture localisation, low statistical power for return to performance analysis.

**Conclusions:**

Thoroughbred racehorses with non‐PSG condylar fractures have a more extensive exercise history than horses with PSG condylar fractures, suggesting differences in fracture aetiopathogenesis.

## Resumo

**Contexto:**

Fraturas condilares são uma das principais causas de morbidade e mortalidade em cavalos de corrida puro‐sangue inglês. As fraturas condilares apresentam uma variedade de configurações que sugerem que pode haver diferenças em suas etiopatogenia.

**Objetivo:**

Determinar se o histórico de exercícios difere com a localização da fratura condilar em uma população de cavalos de corrida puro‐sangue inglês.

**Delineamento do Estudo:**

Análise retrospectiva de dados clínicos e de exercício.

**Métodos:**

O histórico de exercícios de cavalos de corrida puro‐sangue inglês que tiveram reparo de fratura condilar entre 1 de janeiro de 2018 e 28 de fevereiro de 2021 foi comparado entre cavalos de corrida que tiveram fratura localizada radiograficamente dentro da ranhura parasagital (RPS) ou abaxial à RPS (não RPS). Os grupos foram pareados de acordo com a idade, sexo e último evento (corrida ou trabalho cronometrado) para comparação de RPS e não RPS. Além disso, as variáveis de histórico de exercícios de ambos os grupos foram comparadas a uma população de controle específica, cada uma consistindo em três cavalos de corrida de controle com idade e sexo equivalentes combinados com cada cavalo de corrida afetado pelo último evento (corrida ou trabalho cronometrado oficial) antes da fratura.

**Resultados:**

Oitenta e dois cavalos com 84 fraturas (45 RPS, 39 não RPS) atenderam aos critérios de inclusão. A idade não foi diferente entre os grupos (RPS: 3,4 ± 1,3 anos (média ± DP), não RPS: 3,7 ± 1,3, p=0,3). O número de corridas (RPS: 5,3 ± 7,1, não RPS: 11,4 ± 8,9, p<0,001), furlongs totais de corrida (RPS: 38,2 ± 54,7, não RPS: 79,2 ± 64, p=0,003) e número de dias ativos (RPS: 304 ± 224, não RPS: 488 ± 314, p=0,003) antes da fratura foram maiores; enquanto o número médio de repousos foi menor (RPS: 1,0 ± 1,2, não RPS: 0,5 ± 0,7, p=0,02) em cavalos com fratura não RPS. Cavalos com fratura não RPS tiveram mais diferenças em comparação com seu grupo controle respectivo do que cavalos com fraturas RPS. Os resultados após o reparo da fratura não foram diferentes entre os grupos.

**Principais limitações:**

Estudo retrospectivo, uma população regional de cavalos de corrida, imagens bidimensionais e viés inerente potencial para localização de fraturas, baixo poder estatístico para análise de retorno ao desempenho.

**Conclusões:**

Cavalos de corrida puro‐sangue inglês com fraturas condilares não RPS têm um histórico de exercícios mais extenso do que cavalos com fraturas condilares RPS, sugerindo diferenças na etiopatogenia das fraturas.

## 1 INTRODUCTION

Condylar fractures of the third metacarpal and metatarsal bones are one of the leading musculoskeletal injuries resulting in morbidity and mortality in North American Thoroughbred racehorses.^1^, ^2^, ^3^ Clinical and post‐mortem studies have identified the parasagittal groove (PSG) as a common location for condylar fracture and two mechanisms have been proposed to explain the pathophysiology leading to these fractures.^4^, ^5^, ^6^ First, repetitive high peak strain in this region of the condyle leads to fissures which coalesce to form fractures.^4^, ^5^ Second, bone modelling and focal resorption in response to intense exercise create bone density gradients which predispose to fracture.^7^, ^8^, ^9^, ^10^, ^11^

Other clinical studies indicate that condylar fracture can occur abaxial to the parasagittal groove in the palmar/plantar aspect of the condyle.^1^, ^2^, ^11^, ^12^ This region of the condyle can also have areas of subchondral bone damage and focal resorption called Palmar Osteochondral Disease (POD) and these findings are thought to be a separate entity from condylar fracture.^13^, ^14^, ^15^ However, a recent computed tomographic study challenged this idea as 28 of 50 condylar fractures were associated with a lateral subchondral bone injury suggestive of POD.^16^ Similarly, condylar fractures have been documented to originate from lesions that would currently be termed POD lesions.^17^ Given these findings it is conceivable that condylar fractures consist of two distinct fracture populations: those located in the PSG and those located abaxial to the PSG (non‐PSG). Exercise histories of horses that sustained condylar fracture have been evaluated and found to be associated with increased levels of exercise in the months preceding fracture.^18^ However, the location of fracture on the condyle with respect to exercise history has not been examined to determine if fracture location is related to exercise history.

In most instances condylar fractures can be repaired with internal fixation using cortex screws placed in lag fashion followed by a 90–120 day period of convalescence.^1^, ^3^, ^19^ Prognosis for return to racing is favourable but depends upon fracture configuration, presence of articular comminution, concomitant fractures, and pre‐existing degenerative joint disease.^1^, ^3^, ^19^, ^20^ The effect of previous exercise history on outcome following condylar fracture repair has not been evaluated. Similarly, no studies have examined the outcome of horses following condylar fracture repair relative to location of fracture on the condyle. If condylar fractures consist of two distinct populations, outcomes following repair may also differ.

The purpose of the study reported here was to determine if horses with PSG and non‐PSG condylar fractures have distinct exercise histories which might suggest a difference in fracture aetiopathogenesis. This information could possibly be used to inform fracture risk mitigation strategies. Furthermore, identification of any differences in outcome following fracture repair may assist in more accurate prognostication following injury and repair. We hypothesised that in a population of Thoroughbred racehorses presented for condylar fracture repair (1) the prevalence of fractures in the PSG and non‐PSG fractures would not be different, (2) exercise histories of horses with PSG fractures would be different from those of horses with non‐PSG fractures, and (3) horses with PSG fractures would have superior outcomes following surgical repair than horses with non‐PSG fractures.

## 2 MATERIALS AND METHODS

### 2.1 Study design

Radiographic images, exercise history, and outcome of Thoroughbred racehorses following condylar fracture and repair were retrospectively reviewed to identify differences based on fracture location. Data were collected from all Thoroughbred racehorses that presented for condylar fracture of the third metacarpal or third metatarsal bone to one equine surgeon (RC) in California between 1 January 2018 and 28 February 2021. Clinical data were collected for all horses including affected limb, dates of fracture and repair, age, and sex. Race records (Equibase Company LLC) were used to determine number of starts, number of top 3 finishes, and total earnings in the 365 days before fracture and 365 days after fracture repair.

Digital radiographic images of each case were obtained by either the attending surgeon (RC) or referring veterinarian. All images were interpreted by the attending surgeon before surgical intervention for surgical planning. Internal fixation with cortex screws placed in lag fashion was performed under standing sedation or under general anaesthesia if there was fracture displacement or the horse was not amenable to standing surgery. All radiographic studies included a dorsal 20° proximal–palmar/plantarodistal oblique, flexed dorsal 35° proximal–dorsodistal oblique, lateromedial, flexed lateromedial, dorsal 45° lateral–palmar/plantaromedial oblique, and dorsal 45° medial–palmar/plantarolateral oblique.

**FIGURE 1:**
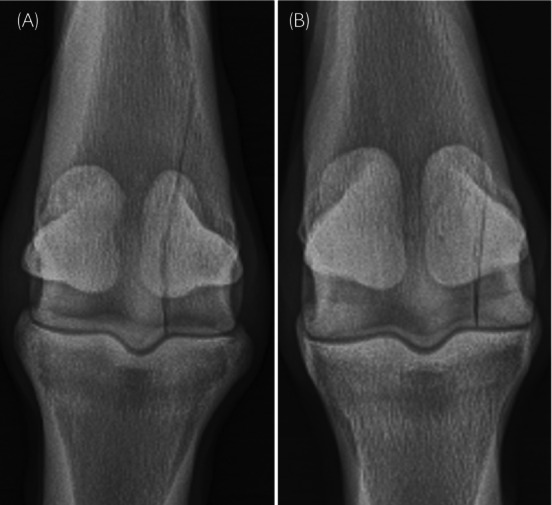
Dorsal 20° proximal–palmarodistal oblique radiographs of (A) 3‐year‐old Thoroughbred with a right forelimb lateral condylar fracture in the parasagittal groove (PSG) and (B) a 4‐year‐old Thoroughbred with a left forelimb lateral condylar fracture abaxial to the PSG.

**FIGURE 2:**
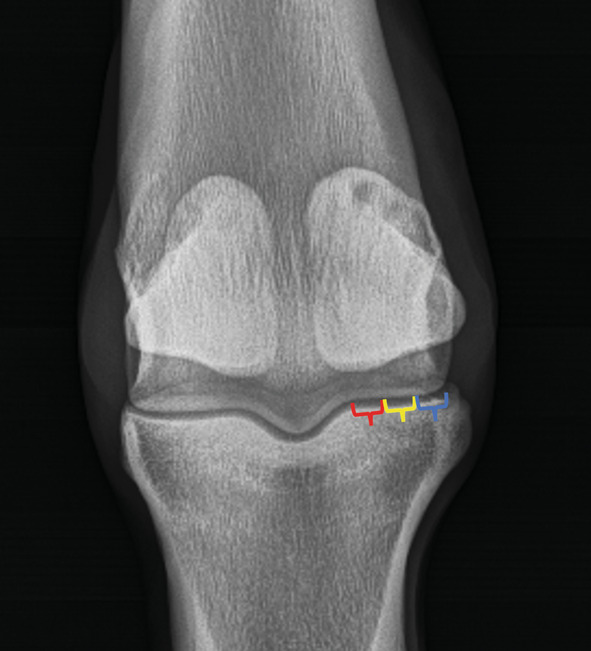
A dorsal 20° proximal–palmarodistal oblique radiograph with a red bracket indicating the position of a non‐PSG axial condyle fracture, a yellow bracket indicating the position of a non‐PSG central condyle fracture, and a blue bracket indicating a non‐PSG abaxial condyle fracture.

Each fracture was subjectively classified as either PSG or non‐PSG, complete or incomplete, and displaced or non‐displaced by consensus between two observers: an equine surgery resident (TB) and a board‐certified radiologist (MS). Fractures were classified as PSG when the radiolucent fracture line intersected the parasagittal groove adjacent to the sagittal ridge (Figure 1A) and as non‐PSG when the line intersected the condylar surface abaxial to the PSG (Figure 1B). The location of the non‐PSG fractures was further described as occurring in the axial, central, or abaxial portions of the condyle by subjectively dividing the flat portion of the condyle into three equally sized segments (Figure 2). Location of non‐PSG fractures on the condyle was not considered in the analysis of exercise history and outcome.

Clinical outcomes were determined by analysis of race records obtained from an online database (Equibase Company LLC). The date of return to racing was the first recorded race start following condylar fracture repair and the number of days from fracture to return to racing was recorded. In all horses that returned to racing, number of starts, number of top 3 finishes, and total earnings in the 365 days following return were recorded. Earnings following return to racing were categorised as positive if earnings in the 365 days following return to racing were greater, and negative if earnings were less, than earnings from the 365 days before fracture.

### 2.2 Data analysis

Descriptive statistics are reported, and the distributions of age, limb (left, right), end (forelimb, hindlimb), and condyle (lateral, medial) were compared between PSG and non‐PSG fractures using a Chi‐square test (SAS 9.4, SAS Institute Inc). The distribution of PSG fracture and non‐PSG fracture prevalence among horses was compared with an even prevalence distribution using a Chi‐square test. The proportions of PSG and non‐PSG horses that returned to performance and that earned more after return to performance than before were compared with a Chi‐square test and post hoc power was determined for proportions of two independent groups (Fisher's exact test) (G*Power Version 3.1.9.7).

Lifetime past performance records of races and timed workouts were obtained (InCompass Solutions, The Jockey Club), truncated to the date of condylar fracture, and reduced to exercise variables consisting of counts of high‐speed events (races, official timed works [works]), layup (period of >60 days without a race or timed work), distances, and rates of events and distances during career and non‐layup (active) portion of career (Table S1), using a custom Matlab program as previously reported.^21^ Exercise history variables were compared between horses with PSG fractures and horses with non‐PSG fractures using a Wilcoxon test for non‐normally distributed data given the difficulty in determining data normality with the relatively small sample sizes (43 PSG, 39 non‐PSG) in this study (SAS 9.4, SAS Institute Inc.).

Additionally, exercise history variables of both groups were each compared with a group‐specific control population, each consisting of three control racehorses of equivalent age and sex matched to each affected racehorse by last event (race or official timed work) before fracture. Variables were compared for PSG and non‐PSG fracture cases between the respective matched controls first using univariable simple logistic regression and conditional logistic regression (SAS 9.4, SAS Institute Inc.). Conditional logistic regression was used to account for undocumented factors associated with racehorse class, that is racehorses are entered in races with other racehorses of similar ability and expected performance without explicit knowledge of the factors that contribute to similar ability and performance. Continuous candidate risk factors were examined for linearity of the odds using the Box–Tidwell transformation. Categorical transformations were examined for continuous variables with non‐linearity of the odds by examining data ranked by quartiles and ranks determined by observed cutpoints. Variables with *p* values ≤0.05 were considered statistically significant. While univariable conditional logistic regression models had better model fit and larger magnitude of odds ratios than univariable simple logistic regression models, there were few variables that had differences in statistical significance (*p* < 0.05) between simple and conditional logistic regression models. Therefore, multivariable models were examined using the simple logistic regression technique in stepwise and backward processes using the AIC criterion for model comparisons. A univariable *p* value <0.20 was used for initial selection of candidate variables for multivariable models. Horse age (days) and sex (female, male) were included in candidate variables. Final model fit was examined using the Hosmer and Lemeshow Goodness‐of‐Fit test and area under the receiver operating curve.

## 3 RESULTS

### 3.1 Signalment

Eighty‐two horses with 84 fractures met the inclusion criteria of the study; one horse had condylar fractures in two separate fetlocks repaired simultaneously, a second horse had two condylar fractures on separate limbs 18 months apart, and the remaining 80 horses had a single condylar fracture. There were 26 fillies and mares, 37 geldings, 17 colts, and sex was unknown for 2 non‐starters. Thirty‐eight fractures were repaired standing under sedation and 46 fractures were repaired under general anaesthesia. Mean age (years) was not significantly different between PSG and non‐PSG horses (Table 1).

**TABLE 1:** Age (mean ± SD), limb, end, and condyle distributions of horses with PSG and non‐PSG fractures.

Fracture	Age (years)	Limb		End		Condyle	
		Left	Right	Forelimb	Hindlimb	Lateral	Medial
PSG	3.4 ± 1.3	20	25	36	9	33	12
Non‐PSG	3.7 ± 1.3	20	19	29	10	37	2
*p*‐value	0.5	0.7		0.2		0.02	

### 3.2 Fracture distribution

Forty‐three horses had PSG fracture and 39 horses had non‐PSG fracture; the distribution of fractures was not different from that of a hypothetical evenly distributed fracture prevalence (41 PSG, 41 non‐PSG) (Chi‐square *p* = 0.6). Horses with two fractures had all PSG fractures for a total of 45 PSG fractures. Thirty‐nine non‐PSG fractures had 31 in the axial third of the lateral condyle, 6 in the central third of the lateral condyle, and 2 in the axial third of the medial condyle. The distributions of PSG and non‐PSG fractures were not significantly different with respect to end (forelimb, hindlimb) or limb (left, right) (Table 1). While both PSG and non‐PSG fractures were most prevalent in the lateral condyle, medial condylar fractures were disproportionately higher in PSG fractures (Table 1).

### 3.3 Exercise history comparison

**FIGURE 3:**
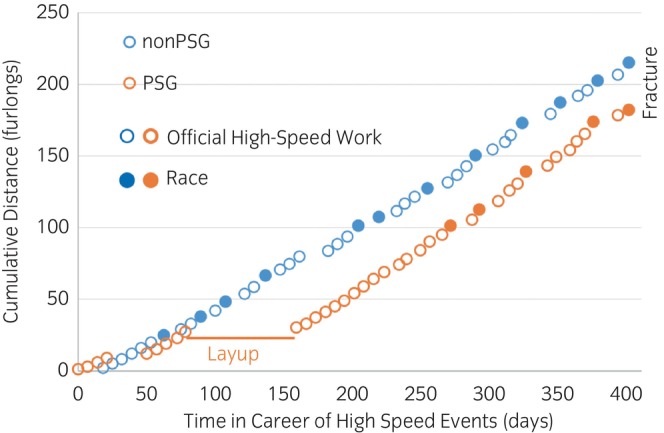
Line plots demonstrating a typical exercise history of a horse with a parasagittal groove (PSG) fracture and of a horse with a non‐PSG fracture. The exercise histories of these two horses were objectively selected based on most closely matching the median number of races, works, and layups for PSG and non‐PSG horses. Races (filled circles), official timed high‐speed works (open circles), layups, and times of fracture are illustrated over time. With each event (race or official timed work), the number of furlongs the horse exercised in that event is added to the number of furlongs exercised in previous events.

Horses with non‐PSG fracture exercised more extensively over the course of their career while horses in the PSG group spent more time in layup but had more works during active periods (Table 2, Figure 3). Median number of races, works, and race and work distances were all 1.2–2.3 times significantly greater for the horses with non‐PSG fracture. All horses that fractured without having raced (*n* = 13) had PSG fracture. Horses with non‐PSG fracture had on average 4 more races per year and 57% less time between races than those with PSG fracture, including 44% less time during active periods (which do not include layups). However, the number of active days was 32% higher and the number of layups was 50% lower in the horses with non‐PSG fracture. The number of works per year during active periods was 18% higher in horses with PSG fracture than in horses with non‐PSG fracture. Similarly, the active work distance rate was 15% higher in the horses with PSG fracture. The number of high‐speed furlongs in the year before fracture were 12–82% higher in the 2–12 months before fracture in horses with non‐PSG fracture. Following the first event in the horse's career there was no difference in the total number of high‐speed furlongs in the first 4 months of training between the two fracture groups. However, from 6 months to 1 year following the first event in the horse's career the horses with non‐PSG fracture had 59%–92% greater numbers of total high‐speed furlongs.

**TABLE 2:** The exercise histories of horses that sustained condylar fracture in the parasagittal groove (PSG) compared with horses that sustained fracture outside the PSG (non‐PSG).

Variable	*n* (case, control)	Non‐PSG		PSG		*p*‐value
		Mean ± SD	Median (Q1, Q3)	Mean ± SD	Median (Q1, Q3)	Wilcoxon *p*‐value
Signalment						
Age (days)	82 (39, 43)	1431 ± 441	1282 (1073, 1840)	1342 ± 433	1220 (986, 1707)	0.3
Age (years)	82 (39, 43)	3.7 ± 1.3	3 (3, 5)	3.4 ± 1.3	3 (2, 4)	0.3
Career						
Career length (days)	82 (39, 43)	577 ± 425	371 (275, 868)	480 ± 408	331 (117, 837)	0.1
Active days (days)	82 (39, 43)	488 ± 314	328 (259, 731)	304 ± 224	249 (109, 439)	**0.009**
Races (#)	82 (39, 43)	11.4 ± 8.9	9 (5, 15)	5.3 ± 7.1	4 (1, 7)	**<0.001**
Works (#)	82 (39, 43)	40.9 ± 27.0	30 (21, 66)	28.3 ± 22.2	23 (10 45)	**0.02**
Events (#)	82 (39, 43)	52.3 ± 34.4	39 (26, 86)	33.6 ± 26.3	30 (11, 52)	**0.01**
Race distance (F)	82 (39, 43)	79.2 ± 64.0	59 (32, 110)	38.2 ± 54.7	27 (4.5, 45.5)	**<0.001**
Work distance (F)	82 (39, 43)	174.3 ± 123.1	120 (80, 262)	120.6 ± 101.8	103 (39, 182)	**0.03**
Event distance (F)	82 (39, 43)	253.4 ± 178.2	179.5 (111, 420)	158.9 ± 138.0	141 (44.5, 238)	**0.01**
Between races (days)	68 (39, 29)	76.8 ± 60.0	56.0 (43.9, 92.8)	143.5 ± 86.6	129.9 (82.8, 168.6)	**<0.001**
Between works (days)	80 (39, 41)	14.6 ± 5.5	13.2 (11.0, 16.8)	20.5 ± 17.7	14.8 (11.1, 22.2)	0.2
Between events (days)	80 (39, 41)	11.1 ± 3.2	10.6 (9.0, 11.9)	16.2 ± 9.9	13.7 (10.4, 18.5)	**0.009**
Layup						
Layups (#)	82 (39, 43)	0.5 ± 0.7	0 (0, 1	1.0 ± 1.2	1 (0, 2)	**0.05**
Layup time (days)	82 (39, 43)	90 ± 139	0 (0, 172)	176 ± 221	0 (0, 331)	**0.06**
Mean layup time (days)	82 (39, 43)	70 ± 106	0 (0, 172)	97 ± 99	81 (0, 184)	0.1
Career in layup (%)	82 (39, 43)	9.5 ± 13.2	0 (0, 19.9)	24.9 ± 25.4	27.1 (0, 44.1)	**0.005**
Time since last layup (days)	82 (39, 43)	390 ± 203	326 (224, 497)	172 ± 121	166 (74, 248)	**<0.001**
Events since last layup (#)	82 (39, 43)	42 ± 23	37 (25, 54)	19 ± 14	14 (9, 30)	**<0.001**
Slope after the last layup	40 (16, 24)	15 ± 3.8	14.8 (12.1, 18.1)	14.1 ± 5.3	16 (9.6, 16.8)	0.8
Rates						
Races (#/year)	82 (39, 43)	7.4 ± 2.5	7.4 (5.5, 9.2)	3.4 ± 2.7	3.1 (1.3, 5.5)	**<0.001**
Works (#/year)	82 (39, 43)	28.4 ± 7.5	28.5 (22.1, 34.1)	26.1 ± 13.1	24.7 (17.7, 34.4)	0.2
Events (#/year)	82 (39, 43)	35.8 ± 7.9	35.5 (31.8, 42.3)	29.4 ± 13.8	26.8 (19.7, 37.2)	**0.006**
Distance per race (F)	72 (39, 33)	6.7 ± 0.9	6.5 (6.0, 7.3)	6.8 ± 1.0	6.8 (6.0, 7.6)	0.5
Distance per work (F)	82 (39, 43)	4.2 ± 0.3	4.2 (3.9, 4.4)	3.9 ± 0.7	4.1 (3.6, 4.3)	0.2
Distance per event (F)	82 (39, 43)	4.7 ± 0.4	4.7 (4.4, 5.1)	4.3 ± 0.9	4.3 (3.9, 4.8)	**0.007**
Career race distance rate (F/months)	82 (39, 43)	4.1 ± 1.4	4.1 (3.0, 5.1)	1.9 ± 1.6	1.6 (0.7, 2.8)	**<0.001**
Career work distance rate (F/months)	82 (39, 43)	9.7 ± 2.8	10.0 (7.8, 11.1)	8.5 ± 4.4	8.2 (4.8, 11.8)	0.2
Career event distance rate (F/months)	82 (39, 43)	13.8 ± 3.1	14.0 (11.6, 16.5)	10.4 ± 5.0	9.8 (7.2, 13.6)	**<0.001**
Active rates						
Races (#/year)	82 (39, 43)	8.2 ± 2.6	8.4 (6.1, 10.1)	4.7 ± 3.9	4.7 (2.5, 6.4)	**<0.001**
Works (#/year)	81 (39, 42)	31.1 ± 6.1	30.8 (26.7, 35.4)	34.6 ± 12.0	36.3 (29.5, 41.2)	**0.02**
Events (#/year)	81 (39, 42)	39.3 ± 5.5	39.4 (35.0, 43.5)	39.4 ± 11.8	41.9 (35.2, 45.4)	0.6
Active career race distance rate (F/months)	82 (39, 43)	4.5 ± 1.6	4.7 (3.2, 5.5)	2.7 ± 2.4	2.7 (1.2, 3.8)	**<0.001**
Active career work distance rate (F/months)	81 (39, 42)	10.7 ± 2.5	10.9 (9.1, 11.9)	11.3 ± 4.2	12.5 (8.6, 14.1)	**0.04**
Active career event distance rate (F/months)	81 (39, 42)	15.2 ± 2.6	15.1 (13.3, 17.0)	14.1 ± 4.7	14.9 (12.4, 17.1)	0.6
Between races active (days)	68 (39, 29)	69.0 ± 55.5	48.3 (39.6, 73.1)	94.5 ± 51.7	86.7 (58.3, 107.8)	**0.002**
Between works active (days)	80 (39, 41)	12.7 ± 2.8	12.0 (10.9, 14.0)	13.9 ± 9.6	10.4 (9.1, 12.8)	**0.02**
Between events active (days)	80 (39, 41)	9.7 ± 1.4	9.4 (8.6, 10.8)	11.6 ± 8.0	9.0 (8.3, 10.8)	0.5
Activity before fracture						
Slope at fracture (f/month)	80 (39, 41)	16.1 ± 4.5	16.1 (12.7, 19.4)	14.2 ± 5.8	15.7 (9.9, 17.5)	0.2
Time between fracture and previous event (days)	82 (39, 43)	11.5 ± 9.4	8 (6, 14)	16.3 ± 19.9	8 (7, 22)	0.6
1 month before fracture (F)	82 (39, 43)	16.8 ± 6.8	16.5 (12, 21)	13.4 ± 7.9	15 (7, 20.5)	0.1
2 months before fracture (F)	82 (39, 43)	33.1 ± 8.6	32.5 (27, 38)	25.9 ± 13.5	29 (17, 35)	**0.02**
4 months before fracture (F)	82 (39, 43)	64.7 ± 15.2	64 (55, 74.5)	47.3 ± 24.2	51 (33, 65)	**0.001**
6 months before fracture (F)	82 (39, 43)	94.4 ± 22.3	91 (77.5, 107.5)	65.9 ± 36.6	65 (40, 99)	**0.001**
8 months before fracture (F)	82 (39, 43)	119.2 ± 30.2	118 (98.5, 139)	78.3 ± 48.4	71.5 (41.5, 125)	**<0.001**
10 months before fracture (F)	82 (39, 43)	139 ± 40.5	142.5 (108, 168)	86.2 ± 55.6	81 (43, 130)	**<0.001**
1 year before fracture (F)	82 (39, 43)	154.2 ± 51.6	161 (108, 190)	93.6 ± 61.3	88.5 (44.5, 148)	**<0.001**
Month 2 (F)	82 (39, 43)	16.3 ± 4.9	17 (13, 20.5)	12.5 ± 7.5	13 (7, 18.5)	**0.02**
Month 3 and 4 (F)	82 (39, 43)	31.6 ± 9.3	32.5 (24, 37)	21.4 ± 15	21 (7, 35.5)	**0.004**
Month 5 and 6 (F)	82 (39, 43)	29.7 ± 10.7	29 (24.5, 36)	18.5 ± 16.7	15 (0, 36)	**0.01**
Month 1 minus 2 (F)	82 (39, 43)	0.5 ± 8.1	0.5 (−5.5, 6)	1 ± 7.6	1.5 (−3.5, 5.5)	0.8
Activity at career beginning						
Slope at start	77 (37, 40)	11.3 ± 4.8	11.6 (7.6, 15.0)	11.2 ± 4.8	11.4 (7.8, 14.1)	0.9
1 month after first event (F)	82 (39, 43)	11.3 ± 6.2	11 (6, 16)	11 ± 5.2	11 (8, 15)	0.9
2 months after first event (F)	82 (39, 43)	22.2 ± 11.2	23 (13, 30.5)	21.7 ± 11.7	23 (9, 13)	0.8
4 months after first event (F)	82 (39, 43)	47.6 ± 20.7	50.5 (33.5, 67)	40.2 ± 23.8	43 (18, 58)	0.1
6 months after first event (F)	82 (39, 43)	72.8 ± 29.3	77 (54.5, 99.5)	51.9 ± 34.3	48.5 (24, 74)	**0.004**
8 months after first event (F)	82 (39, 43)	94 ± 39.7	99.5 (70.5, 127.5)	61.5 ± 42.5	58 (29, 87)	**<0.001**
10 months after first event (F)	82 (39, 43)	111.9 ± 46.3	111 (86.5, 156)	70.5 ± 50.9	62.5 (30.5, 97.5)	**<0.001**
1 year after first event (F)	82 (39, 43)	128 ± 48.8	126 (87.5, 173)	81.7 ± 58.6	66 (43, 125.5)	**<0.001**

*Note*: Variables with *p* < 0.05 are listed in bold type.

### 3.4 Exercise history comparison to age and sex matched controls

#### 3.4.1 PSG fracture versus controls

The horses with fracture in the PSG had few small differences from their age, sex, and event‐matched controls (Table S2). The only significant differences in median values for exercise history between the PSG and control groups were that PSG horses had 17% fewer total high‐speed furlongs in the month before fracture, slightly fewer (1%) works and events during active periods, slightly greater (1%) time between works during total career and active periods and 14% greater time between fracture and the previous event than the control horses.

Multivariable analysis resulted in four variables associated with the PSG outcome, all relative to the active (non‐layup) portion of a horse's career (Table 3). Collectively, odds of having a PSG lateral condylar fracture were highest when the interval between races was between 73 and 110 days, and increased with increasing time between works and with increasing number of works per year; and decreased with increasing high‐speed distance during the month before fracture. Because works, races, events, and distances, and rates of works, races, events, and distances are strongly (0.5 < *r* ^2^ < 0.94) correlated (Table S3), other closely related variables could be useful in differentiating PSG horses from control horses. Days between races during the active portion of career are strongly negatively correlated with numbers and rates of races and race distances. Similarly, days between works during the active portion of career are strongly negatively associated with number of works and events per year, and work furlongs per month, all during the active portion of a career. Distance 1 month before fracture is strongly positively correlated with numbers and distances of races, works, and events; days in career, active portion of career, since last layup and last event; and distances in the last 4–12 months before fracture and 1 year after first event. Number of works during the active portion of career is strongly positively associated with work furlongs and events in the active portion of career and negatively associated with days between works during the active portion of career.

**TABLE 3:** Variables retained in the final multivariable model examining the association between exercise history variables and the PSG outcome for 29 PSG fracture cases and 83 controls.

Variable	Odds ratio		
	OR	95% CI	*p*‐value
Between races active (days)			0.03
20–48	ref		
>48–73	0.65	0.12–3.29	0.6
>73–110	3.47	0.88–16.01	0.07
>110–345	0.87	0.17–4.49	0.8
Between works active (days)	1.34	1.07–1.85	0.006
Distance 1 month before fracture (f)	0.92	0.84–1.00	0.06
Works active (#/year)	1.19	1.03–1.43	0.01

*Note*: Global likelihood ratio test *p*‐value = 0.004, area under the ROC curve = 0.7536. Hosmer and Lemeshow Goodness‐of‐Fit *p* = 0.1229.

#### 3.4.2 Non‐PSG fracture versus controls

In contrast to the comparison of PSG fracture and controls, exercise history before fracture was significantly different between non‐PSG horses and their respective control horses for many variables (Table S4). Median active career length total race distance, total career distance rate, and active career race distance rate were 4%, 49%, 31%, and 46% greater, respectively, in the non‐PSG group than the control group. Non‐PSG horses had greater number of races and works per year, both during entire career (40% and 4% greater, respectively), and 29% greater number of races per year and 14% fewer number of works per year during the active portion of their careers. The median number of days between races and between events were 32% and 7% lower in non‐PSG horses. The median number of layups, days in layup, and proportion of layup time during career were zero in non‐PSG horses and 1, 64 days, and 14% of career in control horses. Similarly, non‐PSG horses had 57% longer time and 61% more events since the last layup. In months 4–10 before fracture non‐PSG horses had significantly more (11%–31%) high‐speed furlongs than the control horses; but 15% marginally insignificant fewer furlongs in the month before fracture (*p* = 0.09). Non‐PSG horses also had 21%–32% greater high‐speed furlongs 6–12 months after their first event (official timed work or race) when compared with control horses. Non‐PSG horses also had 8% and 32% greater number of furlongs in the 3rd and 4th and the 5th and 6th month before fracture, while also having a smaller increase in high‐speed furlongs in the last month compared with the second to last month before fracture.

Multivariable analysis resulted in four variables associated with the non‐PSG outcome (Table 4). Odds of having a non‐PSG lateral condylar fracture increased with increasing number of works per year of total career and number of high‐speed furlongs in the 6 months before fracture; and decreased with increasing work distance rate (f/mo) during the active portion of career and with increasing number of high‐speed furlongs in the month before fracture. Because works, races, events, and distances, and rates of works, races, events, and distances are strongly (0.5 < *r* ^2^ < 0.99) correlated (Table S5), other closely related variables could be useful in differentiating PSG horses from control horses. In general, number of works and work distances are highly correlated with number of events and event distances and negatively correlated with layup variables and days between works and events. However, high‐speed furlong distance in the last month before fracture is only strongly correlated with exercise intensity before fracture (slope before fracture) and distance in the last 2 months before fracture, and high‐speed furlong distance 6 months before fracture is only strongly correlated with high‐speed furlongs in the 4–12 months before fracture.

**TABLE 4:** Variables retained in the final multivariable model examining the association between exercise history variables and the non‐PSG outcome for 39 non‐PSG fracture cases and 117 controls.

Variable	Odds ratio		
	OR	95% CI	*p*‐value
Works (#/year)			
10–21	ref		
>21–28	8.76	2.15–44.77	0.001
>28–35	12.47	2.85–70.32	<0.001
>35–68	12.89	1.99–107.01	0.006
Active career work distance rate (f/month)	0.56	0.39–0.75	<0.001
Distance 1 month before fracture (f)	0.87	0.79–0.95	0.002
Distance 6 months before fracture (f)	1.06	1.03–1.10	<0.001

*Note*: Global likelihood ratio test *p*‐value <0.001, area under the ROC curve = 0.8444. Hosmer and Lemeshow Goodness‐of‐Fit *p* = 0.7141.

### 3.5 Outcome

Return to racing was not significantly different (*p* = 0.2) between PSG horses (28/38, 73.7%) and non‐PSG horses (23/39, 59.0%). Proportion of horses that had greater earnings after return to racing than before condylar fracture was not significantly different (*p* = 0.3) between PSG horses (18/28, 64.3%) and non‐PSG horses (11/23, 47.8%). However, post hoc statistical power for these tests was low (0.19–0.22).

## 4 DISCUSSION

This study evaluated the relationships between location of metacarpal or metatarsal fracture on the condyle with exercise history before condylar fracture, and race performance outcomes following fracture repair. The first hypothesis that the prevalence of horses with PSG and non‐PSG fractures presented for surgical repair would not be different was accepted as 45 PSG fractures and 39 non‐PSG fractures were presented to one surgical referral practice for repair during the 3‐year period of the study. The second hypothesis was accepted as horses with PSG fractures and horses with non‐PSG fracture had significantly different exercise histories before fracture. Horses with non‐PSG fractures had significantly more races, race furlongs, works, work furlongs, fewer layups, greater high‐speed furlongs in the months preceding fracture, and greater high‐speed exercise intensity early in their career than horses with PSG fracture. Further, horses with non‐PSG fracture had more numerous exercise history variables significantly different from event matched control horses than PSG horses. The final hypothesis was not accepted as significant differences were not observed between the proportions of PSG and non‐PSG horses that returned to racing and had greater earnings following their return to racing.

The proportion of fractures that occurred in the forelimb (77%) or hindlimb (23%) and lateral (83%) or medial (17%) in the current study is comparable to previous studies from North American and international populations of racing Thoroughbred horses.^22^ All fractures in the current study occurred in the parasagittal orientation either in the PSG or abaxial to the PSG on the condyle, similar to previous descriptions.^2^, ^12^ In studies of European Thoroughbreds there was an over‐representation of right‐sided condylar fractures but meta‐analysis of multiple studies found that right and left appear similarly affected and direction of racing did not have an apparent effect on condylar fracture distribution.^12^, ^22^, ^23^ Location of the articular condylar component of condylar fracture has been examined in both post‐mortem and clinical studies. In the clinical studies, between 40% and 56% of fractures are located on the condyle in a position consistent with the PSG.^1^, ^2^, ^12^ This finding is similar to the current study as 53% (45/84) of the fractures were PSG fractures. Also similar to the current study, non‐PSG fractures rarely occurred in the most abaxial portion of the condyle and most commonly occurred in the axial or central regions of the condyle.^2^, ^12^ In other clinical studies, and many post‐mortem studies, condylar fractures are described as being located almost exclusively in the PSG.^6^, ^7^, ^8^, ^9^, ^10^, ^24^ The discrepancy between studies concerning fracture location suggests differences in the populations of horses studied. Variables not examined in these studies such as differences in training or racing surface, racehorse management, veterinary practice, and training techniques may account for these differences. Attempts to understand the difference in location of condylar fractures on the condyle in different groups of Thoroughbred racehorses should be the focus of future research.

There were marked differences in the exercise history of horses that sustained PSG fracture compared with horses that sustained non‐PSG fracture. Horse age and career length were not significantly different between PSG and non‐PSG fracture horses, but horses with non‐PSG fractures had significantly more races, works, and total officially recorded high‐speed furlongs than horses with PSG fractures. Differences were pronounced in the 2–12 months before fracture with non‐PSG horses have 12%–93% greater high‐speed furlongs than PSG horses, with the highest discrepancy between non‐PSG and PSG horses in the 5th and 6th months before fracture. PSG horses also had greater high‐speed furlong distances in the 4–12 months after their first event (official timed work or race), although for some horses this period of their career may have overlapped with the period before fracture. PSG horses had limited time to recover from exercise bouts with fewer layup periods and time.

When a comparison was made between PSG horses and their respective control horses, it was apparent that it would be difficult to identify horses at risk for PSG fracture on the basis of exercise variables alone. Median differences between PSG horses and control horses were small. It is notable that in addition to races, exercise during official timed work is relevant to the odds of PSG fracture. Another variable that was interesting was the lower odds associated with greater high‐speed furlongs in the month before fracture. Horses with three additional high‐speed furlongs (observed difference between median values for PSG and control horses) had lowered risk to 78% for PSG fracture. We speculate that horses with pending PSG fracture are displaying some behaviour that influences trainers to decrease horse workload before the subsequent event precipitates fracture. It is also notable that horses with 73–110 days between races had over three times greater odds of PSG fracture, with lesser or more days associated with lower odds. Throughout training the rate of microdamage accumulation is competing with the rates of microdamage removal and adaptive modelling. Further research into the relationships of the timing associated with the acquisition of exercise‐induced microdamage and removal of microdamage with recovery after exercise events is needed.

In contrast to the exercise relationships between PSG horses and their respective control horses, horses with non‐PSG fracture had marked exercise contrasts with their event‐, sex‐, and age‐matched control horses. Similar to the differences between PSG and non‐PSG horses, a large number of variables were statistically different and the magnitudes of the differences were large. Observed findings are consistent with long periods of uninterrupted training and racing with less opportunity for recovery from the intense exercise bouts. Extensive number of works was apparent with an increasing number of works per year contributing the highest odds (8 to >12) for non‐PSG fracture in multivariable analysis, with increasing rate of work furlongs per month during the active portion of career decreasing the odds by 0.06 when considering the differences in median values of non‐PSG horses and controls. The high‐speed furlong distance 6 months before fracture increased odds by 0.76 when adjusted for the difference in median values between non‐PSG fracture and controls. This finding is consistent with excessive workload, likely leading to the inability for bone tissue to recover from accumulated microdamage. Increasing high‐speed furlong distance 1 month before fracture decreased odds by 0.3, consistent similar to PSG horses with the possibility that pending non‐PSG fracture horses are displaying some behaviour that influences trainers to decrease horse workload before a subsequent event precipitates fracture.

It is well documented that resorption and modelling of subchondral bone occur in Thoroughbred racehorses in response to training and has been documented to be associated with increased racing exposure.^14^, ^25^, ^26^, ^27^ Based on the difference in exercise history of the two fracture groups in the current study, non‐PSG fractures may be associated with more subchondral bone modelling and resorption than PSG fractures. The palmar aspect of the condyle abaxial to the PSG is well known to be a predilection site for subchondral bone pathology, potentially leading to POD. The site where POD lesions occur is similar to the sites where the majority of the non‐PSG fractures in the current study were located, that is the axial or central portions of the condyle. Fracture abaxial to the PSG may be related to such subchondral bone injuries, or POD lesions, as recently suggested in a clinical computed tomography study.^16^ It is interesting to speculate that the highest discrepancy in exercise intensity observed between non‐PSG and PSG horses in the 5th and 6th months before fracture could initiate a series of microscopic events in the condyle that induce an inability of the microdamage accumulation to be repaired. Future studies should examine more closely the relationship between exercise history, subchondral bone modelling and resorption in the condyle, POD, and condylar fracture location.

In contrast to horses with non‐PSG fractures, PSG fractures occurred in horses exposed to a less extensive exercise regimen and therefore may have less subchondral bone modelling and resorption. A recent clinical study using Positron Emission Tomography (PET) identified signs suggestive of bone metabolism in the palmar/plantar condyle suggestive of POD in only one of eight horses with PSG fracture.^28^ In accordance with this finding two post‐mortem studies have identified lower levels of palmar osteochondral disease in horses with catastrophic condylar fractures.^23^, ^29^ Collectively, the findings from these studies and the current study suggest that subchondral bone modelling and resorption may have a lesser role in the development of PSG fractures than non‐PSG fractures. Therefore, PSG fractures may be related to insufficient adaptation to training and/or bone density gradients in this region.^8^, ^11^ In support of this theory, PSG horses had more workouts when in active periods but spent more time in layup and had fewer furlongs in the month before fracture and more time between works than the age‐matched controls. Similarly, these findings could indicate that the exercise regimen of PSG horses may not allow for sufficient recovery after official timed works to facilitate adaptation of the distal condyle, thus predisposing to fracture. Developmental abnormalities and/or genetic predisposition may also be a possible factor for these fractures as all 13 horses who suffered a condylar fracture before their first start were in the PSG category.^30^

Although no significant difference was identified in return to performance and earnings after return to racing between horses with PSG or non‐PSG fractures, it is possible that a difference in these groups was not detected because of low statistical power. It is notable that more horses with PSG fracture returned to racing and had greater earnings after returning to racing (73.7%, 64.3%) than those with non‐PSG fracture (59.0%, 47.8%). Previous studies have identified that horses with pre‐existing disease in the fetlock have reduced prognosis following condylar fracture repair and because horses with non‐PSG fracture in the current study have a more extensive exercise history, it is possible they have more pre‐existing disease and their prognosis would be reduced.^1^, ^3^, ^19^, ^20^ However, in another study, subchondral bone injury did not affect outcomes in horses with concurrent subchondral bone injury and condylar fracture.^16^ The findings of these studies suggest that more studies with a larger sample size are needed to fully elucidate the effects of pre‐existing disease and POD on outcome following condylar fracture.

Limitations of the current study include its retrospective nature and low numbers of cases, specifically with regard to the return to performance data. Additionally, data on any arthroscopic assessment of the fetlock joint during fracture repair were not assessed. A second limitation is the identification of fracture location based on two‐dimensional radiographs, which is inferior to three‐dimensional imaging, such as CT, which would allow for a more accurate assessment of fracture location. A third limitation is no direct assessment of POD was performed which would have allowed for the identification of relationships between condylar fracture location with POD and exercise history. Finally, inherent unconscious bias is possible as fracture location was identified by consensus of a single radiologist and an equine surgery resident. Ideally, multiple radiologists would have examined the radiographs to ensure accurate identification of fracture location.

In conclusion, the results of this study demonstrate that metacarpal or metatarsal condylar fractures in Thoroughbred racehorses can be classified into two separate populations, those located in the PSG and those that occur abaxial to the PSG. Horses with these two fractures have significantly different exercise histories suggesting a difference in fracture aetiopathogenesis for the two fracture sites.

## FUNDING INFORMATION

None.

## CONFLICT OF INTEREST STATEMENT

Mathieu Spriet and Susan Stover have an affiliation with LONGMILE Veterinary PET Imaging as unpaid scientific volunteer. The remaining authors declare no competing interests.

## AUTHOR CONTRIBUTIONS

**Thomas C. Bergstrom:** Conceptualization; data curation; formal analysis; investigation; methodology; project administration; resources; software; supervision; validation; visualization; writing – original draft; writing – review and editing. **Mathieu Spriet:** Conceptualization; data curation; formal analysis; investigation; methodology; project administration; resources; software; supervision; validation; visualization; writing – original draft; writing – review and editing. **Ryan Carpenter:** Conceptualization; data curation; formal analysis; investigation; methodology; project administration; resources; software; supervision; validation; visualization; writing – original draft; writing – review and editing. **Kevin L. Jacques:** Data curation. **Susan M. Stover:** Conceptualization; data curation; funding acquisition; investigation; methodology; project administration; resources; software; supervision; validation; visualization; writing – original draft; writing – review and editing.

## DATA INTEGRITY STATEMENT

Thomas C. Bergstrom had full access to all the data in the study and takes responsibility for the integrity of the data and the accuracy of the data analysis.

## ETHICAL ANIMAL RESEARCH

Research ethics committee oversight not required by this journal: retrospective study of clinical records.

## INFORMED CONSENT

Explicit owner consent for animals' inclusion in the study was not stated.

### PEER REVIEW

The peer review history for this article is available at https://www.webofscience.com/api/gateway/wos/peer-review/10.1111/evj.14091.

## Supporting information


**Table S1.** Racehorse injury variable definitions.

**Table S2.** Comparison of exercise history variables between horses with fractures inside the parasagittal groove (PSG) and three age‐ and sex‐matched control horses from each injured horse's last event.

**Table S3.** PSG Control data correlations.

**Table S4.** Comparison of exercise history variables with fracture abaxial to the parasagittal groove (non‐PSG) and three age‐ and sex‐matched control horses from each injured horse's last event.

**Table S5.** Non‐PSG control data correlations.

## Data Availability

The data that support the findings of this study are available from the corresponding author upon reasonable request: Open sharing exemption granted by editor for this descriptive retrospective clinical report.
