# Supplementary material for: Condylar fracture location is correlated to exercise history in Thoroughbred racehorses
Source: Equine Vet J. 2024 Apr 7;57(1):76–86. doi: 10.1111/evj.14091 (PMC11616956; doi:10.1111/evj.14091)
Supplement: Supplementary file 2 — Table S2. Comparison of exercise history variables between horses with fractures inside the parasagittal groove (PSG) and three age‐ and sex‐matched control horses from each injured horse's last event. [file EVJ-57-76-s003.pdf]

Equine Veterinary Journal BEVA

Table S2: Comparison of exercise history variables between horses with fracture inside the parasagittal groove (PSG) and three age and sex matched control horses from each injured horse's last event. Variables with P<0.05 are listed in bold type.

		PSG Fracture		Control		Univariable Simple Logistic Regression			
Variable	n (case, control)	Mean ± SD	Median (min, max)	Mean ± SD	Median (min, max)	OR	95% CIs	OR p-value	likelihood ratio p-value
SIGNALMENT									
Age (days)	43, 129	1341.8 ± 433.3	1220 (786-2484)	1333.9 ± 426.1	1213 (746-2510)	1	0.999-1.001	0.9	0.9
Age (years)	43, 129	3.4 ± 1.3	3 (2-7)	3.4 ± 1.3	3 (2-7)	1.015	0.771-1.336	0.9	0.9
CAREER									
Career length (days)	43, 129	480.2 ± 408	331 (34-1652)	491.0 ± 422.5	373 (7-1701)	1	0.999-1.001	0.8	0.9
Active career length (days)	43, 129	304.4 ± 223.7	249 (1-908)	347.7 ± 303.6	259 (7-1368)				
1-498						ref			0.3
498-1368						0.614	0.260-1.452	0.2	
Races (#)	43, 129	5.3 ± 7.1	4 (0-39)	6.3 ± 7.6	3 (0-37)	0.98	0.932-1.131	0.4	0.4
Works (#)	43, 129	28.3 ± 22.2	23 (1-88)	32.3 ± 26.7	25 (1-118)	0.994	0.980-1.008	0.3	0.4
Events (#)	43, 129	33.6 ± 26.3	30 (1-106)	38.7 ± 33.3	28 (2-143)	0.995	0.983-1.005	0.3	0.4
Race Distance (F)	43, 129	38.2 ± 54.7	27 (0-297.5)	45.4 ± 56.8	20 (0-284.5)	0.998	0.991-1.004	0.4	0.5
Work Distance (F)	43, 129	120.6 ± 101.8	103 (3-420)	137.7 ± 119.5	102 (3-510)	0.999	0.996-1.002	0.4	0.4
Events Distance (F)	43, 129	158.9 ± 138	141 (3-561)	183.1 ± 169.4	127 (5-729.5)	0.999	0.997-1.001	0.3	0.4
Between races	29, 84	143.5 ± 86.6	129.9	140.6 ± 127.0	83.6				

(days)			(40.1-377.7)			(34.8-682)					
34-64								ref			0.06
>64-90								2.777	0.638-<br>12.097	0.1	
>90-176								5.881	1.440-<br>24.018	0.01	
>176-682								2.777	0.638-<br>12.097	0.1	
**Between works<br>(days)**	41,<br>126	20.5 ± 17.7	14.8<br>(6-92.4)		15.9 ± 9.7	13.8<br>(7-89.2)		1.027	0.999-<br>1.055	0.5	**0.04**
Between events<br>(days)	41,<br>128	16.2 ± 9.9	13.7<br>(5.3-49)		14.0 ± 7.2	12<br>(6.4-44.6)		1.032	0.992-<br>1.075	0.1	0.1
LAYUP											
Layups<br>(#)	43,<br>129	1.0 ± 1.2	1<br>(0-4)		1.0 ± 1.1	1<br>(0-4)		0.032	0.758-<br>1.403	0.8	0.8
Layup time<br>(days)	43,<br>128	175.7 ± 221.1	81<br>(0-875)		143.5 ± 175.1	103<br>(0-720)		0.001	0.999-<br>1.003	0.3	0.3
Mean Layup time<br>(days)	43,<br>128	97.1 ± 99.3	81<br>(0-331)		82.6 ± 85.6	83.8<br>(0-329)		1.002	0.998-<br>1.006	0.3	0.4
Career in Layup<br>(%)	43,<br>128	24.9 ± 25.4	27.1<br>(0-99.2)		21.2 ± 23.1	19.3<br>(0-83.5)		1.006	0.992-<br>1.021	0.3	0.4
Time since last<br>Layup<br>(days)	43,<br>129	171.6 ± 120.7	166<br>(0-472)		197.5 ± 182.4	130<br>(0-848)					
0-66								ref			0.2
>66-134								1.145	0.413-<br>3.175	0.7	
>134-269								2.239	0.857-<br>5.849	0.1	
>269-848								0.863	0.298-<br>2.500	0.7	
Events since last<br>layup<br>(#)	43,<br>129	18.6 ± 13.7	14<br>(0-56)		21.9 ± 19.7	15<br>(0-96)					
0-7								ref			0.2
>7-15								2.269	0.832-<br>6.187	0.1	
>15-31								1.801	0.670-	0.2	

									4.841		
>31-96								0.928	0.303-2.845	0.8	
Slope after the last layup (f/mo)	24, 67	14.1 ± 5.3	16 (4.3-23.1)		15.2 ± 4.1	15.9 (4.4-25.9)		0.946	0.852-1.050	0.2	0.3
RATES											
Races (#/yr)	43, 129	3.4 ± 2.7	3.1 (0 -10.3)		3.7 ± 3.0	3.5 (0- 10.9)		0.963	0.855-1.086	0.5	0.5
Works (#/yr)	43, 128	26.1 ± 13.1	24.7 (3.1-68.4)		30.8 ± 17.3	27.3 (0.4-91.3)		0.979	0.953-1.003	0.08	0.08
Events (#/yr)	43, 128	29.4 ± 13.8	26.8 (3.1-76.0)		34.5 ± 16.7	31.3 (0.4-91.3)		0.978	0-951-1.001	0.06	0.06
Distance per race (f)	33, 97	6.8 ± 1.0	6.8 (4.5-8.4)		6.8 ± 1.0	7.0 (4.5-8.9)		1.018	0.689-1.520	0.9	0.9
Distance per work (f)	43, 129	3.9 ± 0.7	4.1 (1.6-4.9)		4.0 ± 0.6	4.1 (2.0-4.9)		0.86	0.488-1.555	0.6	0.6
Distance per event (f)	43, 129	4.3 ± 0.9	4.3 (1.6-6.6)		4.3 ± 0.8	4.4 (2.0-6.5)		0.97	0.597-1.396	0.6	0.6
Career race distance rate (f/mo)	43, 129	1.9 ± 1.6	1.6 (0-6.1)		2.1 ± 1.8	1.8 (0-6.9)		0.942	0.764-1.151	0.5	0.5
Career work distance rate (f/mo)	43, 129	8.5 ± 4.49	8.2 (0.8-20)		9.9 ± 4.5	9.3 (0.1-25.7)		0.93	0.852-1.008	0.07	0.07
Career event distance rate (f/mo)	43, 129	10.4 ± 5.0	9.8 (0.8-22.8)		11.9 ± 4.6	11.6 (0.1-25.7)		0.932	0.862-1.004	0.06	0.06
ACTIVE RATES											
Races (#/yr)	43, 129	4.7 ± 3.9	4.7 (0-18.3)		4.8 ± 3.6	4.7 (0-15.4)		0.994	0.903-1.092	0.9	0.9
**Works (#/yr)**	42, 128	34.6 ± 12.0	36.3 (9.9-68.4)		39.6 ± 15.55	36.3 (4.3-91.3)		0.973	0.946-1.001	0.06	**0.04**
**Events (#/yr)**	42, 128	39.4 ± 11.8	41.9 (10.7-76.0)		44.4 ± 13.7	42.5 (13.0-91.3)		0.967	0.935-0.996	0.02	**0.02**
Active career	43,	2.7 ± 2.4	2.7		2.7 ± 2.2	2.3		0.993	0.847-	0.9	0.9

race distance rate (f/mo)	129		(0-11.5)			(0-11.2)			1.155		
Active career work distance rate (f/mo)	42, 128	11.3 ± 4.2	12.5 (2.6-20.0)		12.7 ± 3.9	12.4 (1.1-24.5)		0.917	0.836-1.003	0.05	0.05
Active career event distance rate (f/mo)	42, 128	14.1 ± 4.7	14.9 (2.6-22.8)		15.4 ± 3.6	15.7 (5-24.5)		0.92	0.842-1.004	0.06	0.06
**Between races active (days)**	29, 85	94.5 ± 51.7	86.7 (20.4-219)		94.7 ± 69.5	64.9 (26.1-345)					
20-48								ref			**0.05**
>48-72								0.587	0.146-2.353	0.4	
>72-109								2.979	0.932-9.523	0.06	
>109-345								1	0.279-3.584	1	
**Between works active (days)**	41, 127	13.9 ± 9.6	10.4 (6-49)		11 ± 3.6	10.3 (5.5-32)		1.08	1.020-1.155	0.008	**0.008**
Between events active (days)	41, 129	11.6 ± 8	9 (5.3-49)		9.9 ± 4.6	9 (5.5-42)		1.044	0.986-1.107	0.1	0.1
ACTIVITY BEFORE FRACTURE											
Slope at Fracture (f/mo)	41, 124	14.2 ± 5.8	15.7 (2.1-27.3)		16 ± 5.5	16.3 (3.1-29.5)		0.945	0.885-1.007	0.08	0.08
**Time between fracture and previous event (days)**	43, 129	16.3 ± 19.9	8 (1-117)		16.2 ± 37.7	7 (1-329)					
1-12								Ref			**0.02**
>12-329								2.465	1.156-5.255	0.01	
**1 mo before fracture**	43, 129	13.4 ± 7.9	15 (0-30)		16.4 ± 7.2	18 (0-30.5)		0.949	0.904-0.994	0.02	**0.02**

(f)											
2 mos before fracture (f)	43, 129	25.9 ± 13.5	29 (0-55)		28.2 ± 12.8	30.5 (0-48)		0.987	0.961-1.013	0.3	0.3
4 mos before fracture (f)	43, 129	47.3 ± 24.2	51 (3-90.5)		48 ± 25.6	49.5 (0-93)		0.999	0.985-1.013	0.8	0.8
6 mos before fracture (f)	43, 129	65.9 ± 36.6	65 (3-128.5)		64.4 ± 37.7	66.5 (3-146.5)		1.001	0.992-1.010	0.8	0.8
8 mos before fracture (f)	43, 129	78.3 ± 48.4	71.5 (3-166.5)		78.3 ± 49	71.5 (3-187.5)		1	0.993-1.007	0.9	0.9
10 mos before fracture (f)	43, 129	86.2 ± 55.6	81 (3-189)		90 ± 58.4	86 (3-238.5)		0.999	0.993-1.005	0.7	0.7
1 yr before fracture (f)	43, 129	93.6 ± 61.3	88.5 (3-224)		103.3 ± 68.4	104 (3-282.5)		0.998	0.992-1.003	0.4	0.4
Month 2 (f)	43, 129	12.5 ± 7.5	13 (0-26)		11.9 ± 7.5	14 (0-27)		1.011	0.966-1.061	0.6	0.6
Month 3 and 4 (f)	43, 129	21.4 ± 15	21 (0-47)		19.8 ± 15.9	20.5 (0-49.5)		1.007	0.985-1.029	0.5	0.5
Month 5 and 6 (f)	43, 129	18.5 ± 16.7	15 (0-43)		16.4 ± 16.4	12 (0-53.5)		1.008	0.987-1.029	0.4	0.4
**Month 1 minus 2 (f)**	43, 129	1 ± 7.6	1.5 (-15-17)		4.5 ± 7	5 (-19-20.5)		0.935	0.889-0.981	0.005	**0.005**
ACTIVITY AT CAREER BEGINNING											
Slope at start (f/mo)	40, 123	11.2 ± 4.8	11.4 (2.1-27.3)		11.6 ± 4.7	11.5 (2.5-25.9)		0.983	0.910-1.060	0.6	0.6
1 mo after first event (f)	43, 129	11 ± 5.2	11 (3-24)		11.2 ± 5.1	10 (2-23)		0.995	0.929-1.064	0.8	0.08
2 mos after first event (f)	43, 129	21.7 ± 11.7	23 (3-43)		19.7 ± 11.1	20 (2-49)		1.016	0.985-1.048	0.3	0.3
4 mos after first event	43, 129	40.2 ± 23.8	43 (3-93.5)		36.6 ± 21.9	36 (3-88)		1.007	0.992-1.023	0.3	0.3

(f)											
6 mos after first event (f)	43, 129	51.9 ± 34.3	48.5 (3-128.5)		50.9 ± 31.8	46 (4-122.5)		1.001	0.990-1.012	0.8	0.8
8 mos after first event (f)	43, 129	61.5 ± 42.5	58 (3-171)		63.9 ± 41.8	59.5 (4-167.5)		0.999	0.990-1.007	0.7	0.7
10 mos after first event (f)	43, 129	70.5 ± 50.9	62.5 (3-222.5)		75.4 ± 49.6	66.5 (4-199)		0.998	0.991-1.005	0.5	0.5
1 yr after first event (f)	43, 129	81.7 ± 58.6	66 (3-263.5)		88.2 ± 58.2	76 (4-231.5)		0.998	0.992-1.004	0.5	0.5
