# Supplementary material for: Condylar fracture location is correlated to exercise history in Thoroughbred racehorses
Source: Equine Vet J. 2024 Apr 7;57(1):76–86. doi: 10.1111/evj.14091 (PMC11616956; doi:10.1111/evj.14091)
Supplement: Supplementary file 4 — Table S4. Comparison of exercise history variables with fracture abaxial to the parasagittal groove (non‐PSG) and three age‐ and sex‐matched control horses from each injured horse's last event. [file EVJ-57-76-s002.pdf]

Table S4: Comparison of exercise history variables between horses with fracture abaxial to the parasagittal groove (non PSG) and three age and sex matched control horses from each injured horse's last event. Variables with P<0.05 are listed in bold type.

		Non PSG Fracture		Control		Univariable Simple Logistic Regression			
Variable	n (case, control	Mean ± SD	Median (min, max)	Mean ± SD	Median (min, max)	OR	95% CIs	OR p-value	Likelihood Ratio p-value
SIGNALMENT									
Age (days)	39, 117	1431 ± 441.3	1282 (913-2311)	1429.4 ± 443.8	1289 (859-2391)	1	0.999-1.001	0.9	0.9
Age (yrs)	39, 117	3.7 ± 1.3	3 (2-6)	3.7 ± 1.3	3 (2-6)	0.995	0.749-1.310	0.9	0.9
CAREER									
Career length (days)	39, 117	577.4 ± 425.4	371 (97-1627)	571.1 ± 436.7	400 (16-1598)	1	0.999-1.001	0.9	0.9
**Active career length (days)**	39, 117	487.6 ± 313.7	328 (97-1251)	419.9 ± 319.2	314 (16-1352)				**0.002**
16-210						ref			
>210-321						10.799	2.267-51.447	0.002	
>321-640						6.206	1.259-30.585	0.02	
>640-1352						7.999	1.651-38.753	0.009	
Races (#)	39, 117	11.4 ± 8.9	9 (2-41)	8.4 ± 8.6	5 (0-38)				0.06
0-3						ref			
>3-6						2.285	0.652-8.014	0.1	
>6-13						4.173	1.193-14.595	0.02	
>13-41						3.851	1.123-13.204	0.03	

Works (#)	39, 117	40.9 ± 27	30 (11-111)	37.6 ± 27	31 (3-125)	1.004	0.991-1.018	0.5	0.5
Events (#)	39, 117	52.3 ± 34.4	39 (13-133)	45.9 ± 34.5	35 (3-161)	1.005	0.995-1.015	0.3	0.3
**Race Distance (F)**	39, 117	79.2 ± 64	59 (11.5-250.5)	60.2 ± 65.2	39.5 (0-291.5)				**0.04**
0-43						ref			
>43						2.156	1.020-4.559	0.04	
Work Distance (F)	39, 117	174.3 ± 123.1	120 (43-525)	160.6 ± 120	129 (10-559)	1.001	0.998-1.004	0.5	0.5
Events Distance (F)	39, 117	253.4 ± 178.2	179.5 (55-695.5)	220.9 ± 177.9	162.5 (10-850.5)	1.011	0.999-1.003	0.3	0.1
**Between races (days)**	39, 94	76.8 ± 60	56 (32.8-298)	99.8 ± 72.5	73.9 (30.8-454)				**0.02**
30-50						ref			
>50-68						0.522	0.190-1.433	0.2	
>68-109						0.5	0.183-1.368	0.1	
>109-454						0.166	0.047-0.578	0.004	
Between works (days)	39, 117	14.6 ± 5.5	13.2 (8.6-39.2)	15.5 ± 6.1	13.6 (6.6-40.8)	0.972	0.910-1.038	0.3	0.3
**Between events (days)**	39, 117	11.1 ± 3.2	10.6 (7.8-23.9)	12.9 ± 4.9	11.3 (6.6-31.1)	0.9	0.813-0.997	0.04	**0.02**
LAYUP									
**Layups (#)**	39, 117	0.5 ± 0.7	0 (0-2)	0.9 ± 1.1	1 (0-5)	0.634	0.396-0.947	0.02	**0.02**
**Layup time (days)**	39, 117	89.8 ± 138.9	0 (0-448)	151.2 ± 195.9	64 (0-956)	0.998	0.995-1.000	0.05	**0.02**
Mean Layup time (days)	39, 117	70.4 ± 105.7	0 (0-448)	86.8 ± 100.4	64 (0-511)	0.998	0.994-1.002	0.3	0.3

**Career in Layup (%)**	39, 117	9.5 ± 13.2	0 (0-46.6)	19.5 ± 22.3	14.4 (0-77)				**0.004**
0-30						ref			
>30-77						0.117	0.027-0.551	0.004	
**Time since last Layup (days)**	39, 117	390.1 ± 202.9	326 (97-868)	269.1 ± 243.7	207 (1-1226)				<**0.001**
1-144						ref			
>144-250						13.103	1.586-108.257	0.01	
>250-364						16.889	2.071-137.747	0.008	
>364-1226						26.434	3.284-212.773	0.002	
**Events since last layup (#)**	39, 117	41.6 ± 22.7	37 (13-102)	29.4 ± 25.1	23 (1-131)				**<0.001**
1-16						ref			
>16-25						16.923	2.060-138.995	0.008	
>25-39						13.793	1.671-113.816	0.01	
>39-131						30.908	3.851-248.092	0.001	
Slope after the last layup (f/mo)	16, 59	15 ± 3.8	14.8 (7.5-20.2)	15.3 ± 4.3	16.4 (4.5-21.2)	0.983	0.864-1.127	0.7	0.7
RATES									
**Races (#/yr)**	39, 117	7.4 ± 2.5	7.4 (2.5-12.2)	5.0 ± 3.2	5.3 (0- 12.9)	1.312	1.151-1.520	<0.001	**<0.001**

**Works (#/yr)**	39, 117	28.4 ± 7.5	28.5 (10.2-43.7)	28.4 ± 10.7	27.4 (10.4-68.4)				**0.01**
10-21						ref			
>21-28						4.347	1.278-14.964	0.01	
>28-35						5.467	1.616-18.5	0.006	
>35-68						1.913	0.512-7.153	0.3	
Events (#/yr)	39, 117	35.8 ± 7.9	35.5 (16.1-48.9)	33.4 ± 11	34.5 (12.6-68.4)				0.06
12-25						ref			
>25-34						4.374	1.278-14.964	0.01	
>34-40						3.436	0.987-44.964	0.05	
>40-68						3.436	0.987-44.964	0.05	
Distance per race (f)	39, 102	6.7 ± 0.9	6.6 (5.3-9.2)	6.9 ± 1.1	6.8 (5.0-9.9)	0.833	0.565-1.204	0.3	0.3
Distance per work (f)	39, 117	4.2 ± 0.3	4.2 (3.5-4.8)	4.2 ± 0.3	4.2 (3.1-5.1)	0.976	0.330-2.937	0.9	0.9
Distance per event (f)	39, 117	4.7 ± 0.4	4.7 (4.0-5.7)	4.6 ± 0.5	4.6 (3.3-6.3)				0.4
3.3-4.3						ref			
>4.3-4.6						1.18	0.382-3.646	0.7	
>4.6-4.9						2.032	0.701-5.885	0.1	
>4.9-6.3						2.032	0.701-5.886	0.1	

**Career race distance rate (f/mo)**	39, 117	4.1 ± 1.4	4.1 (1.2-6.8)	2.8 ± 1.8	2.8 (0-7.1)	1.532	1.222-1.965	<0.001	**<0.001**
Career work distance rate (f/mo)	39, 117	9.7 ± 2.8	10.0 (3.8-17.1)	9.7 ± 3.5	9.7 (3.1-19.6)				0.07
3.1-7.2						ref			
>7.2-9.7						3.808	1.213-11.957	0.02	
>9.7-11.9						3.022	0.948-9.632	0.06	
>11.2-19.5						1.755	0.519-5.936	0.3	
Career event distance rate (f/mo)	39, 117	13.8 ± 3.1	14 (7.5-20.2)	12.5 ± 4.0	12.8 (4.0-21.5)				
4.0-9.8						ref			0.2
>9.8-13.1						2.671	0.830-8.602	0.09	
>13.1-16.0						3.022	0.948-9.632	0.06	
>16.0-21.5						2.671	0.830-8.602	0.09	
ACTIVE RATES									
**Races (#/yr)**	39, 117	8.2 ± 2.6	8.4 (2.7-13.6)	6.2 ±3.6	6.5 (0-15.2)	1.204	1.068-1.357	0.002	**0.001**
0-4.8						ref			0.02
>4.8-6.7						2.624	0.734-9.388	0.1	
>6.7-8.9						3.436	0.987-11.964	0.05	
>8.9-15.2						5.467	1.616-18.500	0.006	
**Works (#/yr)**	39, 117	31.1 ± 6.1	30.8 (19.0-43.7)	35.5 ± 8.6	35.0 (17.3-68.4)	0.922	0.869-0.972	0.001	**0.001**
Events (#/yr)	39, 117	39.3 ± 5.5	39.4 (30.2-48.9)	41.7 ± 7.3	40.5 (23.0-68.4)	0.948	0.892-1.002	0.05	0.05
**Active career race distance rate (f/mo)**	39, 117	4.6 ± 1.6	4.7 (1.3-8.5)	3.5 ± 2.2	3.6 (0-9.4)				**0.03**

0-2.5						ref			
>2.5-3.9						3.016	0.856-10.629	0.08	
>3.9-5.2						3.016	0.856-10.629	0.08	
>5.2-9.4						5.467	1.616-18.500	0.006	
**Active career work distance rate (f/mo)**	39, 117	10.7 ± 2.5	10.9 (6.1-17.1)	12.1 ± 2.7	12 (4.7-19.6)	0.809	0.690-0.936	0.003	**0.004**
Active career event distance rate (f/mo)	39, 117	15.2 ± 2.6	15.1 (10.9-20.9)	15.6 ± 2.7	15.9 (7.2-21.5)	0.948	0.829-1.084	0.4	0.4
Between races active (days)	39, 94	69 ± 55.5	48.3 (27.9-275)	73.5 ± 44.8	61.8 (28.8-291)				0.1
27-43						ref			
>43-57						0.879	0.325-2.378	0.7	
>57-79						0.399	0.135-1.181	0.09	
>79-291						0.141	0.140-1.230	0.1	
**Between works active (days)**	39, 117	12.7 ± 2.8	12 (8.6-20.9)	11.4 ± 2.7	10.8 (6.5-22.6)	1.176	1.033-1.338	0.01	**0.01**
Between events active (days)	39, 117	9.7 ± 1.4	9.4 (7.6-12.8)	9.4 ± 1.7	9.3 (6.5-16.7)	1.122	0.906-1.389	0.2	0.2
ACTIVITY BEFORE FRACTURE									
Slope before fracture (f/mo)	39, 116	16.1 ± 4.5	16.1 (7.5-27)	16.4 ± 4.3	16.4 (4.8-26.1)	0.984	0.904-1.071	0.7	0.6

Time between fracture and previous event (days)	39, 117	11.5 ± 9.4	8<br>(1-50)	10.8 ± 16.8	7<br>(1-183)	1.003	0.981-1.025	0.8	0.8
1 mo before fracture<br>(f)	39, 117	16.8 ± 6.8	16.5<br>(0-32)	18.6 ± 5.3	19<br>(4-32)	0.947	0.889-1.009	0.09	0.09
2 mos before fracture<br>(f)	39, 117	33.1 ± 8.6	32.5<br>(11.5-50)	33.3 ± 9.3	34<br>(4-51)	0.998	0.959-1.039	0.9	0.9
4 mos before fracture<br>(f)	39, 117	64.7 ± 15.2	64<br>(34-103)	58.8 ± 19.8	63.5<br>(4-95)	1.018	0.998-1.041	0.08	0.08
**6 mos before fracture<br>(f)**	39, 117	94.4 ± 22.3	91<br>(48.5-148)	78.8 ± 29	82<br>(4-134.5)	1.023	1.008-1.039	0.001	**0.001**
**8 mos before fracture<br>(f)**	39, 117	119.2 ±<br>30.2	118<br>(55-197)	96.2 ± 40.2	96<br>(10-178)				**<0.001**
10-75						ref			
>75-197						18.278	2.418-<br>138.189	0.004	
**10 mos before fracture<br>(f)**	39, 117	139 ± 40.5	142.5<br>(55-244)	110.2 ±<br>49.3	109<br>(10-227)				**0.001**
10-80						ref			
>80-117						7.536	1.543-36-813	0.01	
>117-244						9.074	2.028-40.595	0.003	
1 yr before fracture<br>(f)	39, 117	154.2 ±<br>51.6	161<br>(55-269.5)	123.4 ±<br>58.5	118<br>(10-258.5)				0.06
10-89						ref			

>89-127						2.345	0.719-7.648	0.1	
>127-174						2.04	0.615-6.762	0.2	
>174-270						4.25	1.360-13.276	0.01	
Month 2 (f)	39, 117	16.3 ± 4.9	17 (4-24)	14.7 ± 6.1	16 (0-27)	1.051	0.985-1.128	0.1	0.1
**Month 3 and 4 (f)**	39, 117	31.6 ± 9.3	32.5 (3-53)	25.5 ± 13.4	30 (0-49)	1.046	1.010-1.083	0.01	**0.006**
**Month 5 and 6 (f)**	39, 117	29.7 ± 10.7	29 (0-51)	20 ± 14.1	22 (0-48.5)	1.061	1.027-1.096	<0.001	**<0.001**
**Month 1 minus 2 (f)**	39, 117	0.5 ± 8.1	0.5 (-15.5-15.1)	3.9 ± 6.7	4 (-13-19)	0.935	0.886-0.986	0.01	**0.01**
ACTIVITY AT CAREER BEGINNING									
Slope at start (f/mo)	37, 115	11.3 ± 4.8	11.6 (3.7-20.6)	11.7 ± 4.8	11.3 (2.5-30)	0.983	0.908-1.064	0.6	0.6
1 mo after first event (f)	39, 117	11.3 ± 6.2	11 (1-24)	11.5 ± 5.2	12 (1-23)	0.991	0.927-1.060	0.7	0.7
2 mos after first event (f)	39, 117	22.2 ± 11.2	23 (2-43.5)	22.2 ± 10	24 (1-44.5)	1.001	0.966-1.037	0.9	0.9
4 mos after first event (f)	39, 117	47.6 ± 20.7	50.5 (2-77.5)	43.3 ± 21.4	45 (1-82)	1.01	0.992-1.028	0.2	0.2
**6 mos after first event (f)**	39, 117	72.8 ± 29.3	77 (2-110)	61.1 ± 32.8	63.5 (1-122.5)	1.012	1-1.024	0.05	**0.04**
**8 mos after first event (f)**	39, 117	94 ± 39.7	99.5 (2-152)	77.6 ± 41.8	75.5 (1-173.5)	1.01	1.001-1.019	0.03	**0.03**
**10 mos after first event (f)**	39, 117	111.9 ± 46.3	111 (12-185)	91.6 ± 50.1	91.5 (6-220.5)	1.008	1.001-1.016	0.02	**0.02**

**1 yr after first event (f)**	39, 117	128 ± 48.8	126 (35-211)	105.8 ± 56.6	99.5 (10-273.5)	1.007	1.001-1.014	0.03	**0.03**
